# Preoperative Localization and Surgical Margins in Conservative Breast Surgery

**DOI:** 10.1155/2013/793819

**Published:** 2013-08-05

**Authors:** F. Corsi, L. Sorrentino, D. Bossi, A. Sartani, D. Foschi

**Affiliations:** Surgery Division, Department of Clinical Sciences, L. Sacco Hospital, University of Milan, Via G.B. Grassi 74, 20157 Milan, Italy

## Abstract

Breast-conserving surgery (BCS) is the treatment of choice for early breast cancer. The adequacy of surgical margins (SM) is a crucial issue for adjusting the volume of excision and for avoiding local recurrences, although the precise definition of an adequate margins width remains controversial. Moreover, other factors such as the biological behaviour of the tumor and subsequent proper systemic therapies may influence the local recurrence rate (LRR). However, a successful BCS requires preoperative localization techniques or margin assessment techniques. Carbon marking, wire-guided, biopsy clips, radio-guided, ultrasound-guided, frozen section analysis, imprint cytology, and cavity shave margins are commonly used, but from the literature review, no single technique proved to be better among the various ones. Thus, an association of two or more methods could result in a decrease in rates of involved margins. Each institute should adopt its most congenial techniques, based on the senologic equipe experience, skills, and technologies.

## 1. Introduction

Breast-conserving surgery (BCS) is the treatment of choice for early breast cancer [1, 2]. Various randomized trials have reported this approach to be safe and effective, thus determining a decrease in the adoption of mastectomy as the treatment of choice for early invasive breast cancer [3, 4]. BCS can almost be considered the gold standard of early stage invasive breast cancer treatment, allowing to achieve adequate surgical margins (SM) with an acceptable cosmetic outcome. Some studies have defined the adequacy of SM by its correlation with the locoregional recurrence rate (LRR) [5–14], but the precise definition of an adequate margins width remains controversial [15–17]. However, there is no doubt that obtaining negative margins decreases the risk of local recurrence [1]. Some clinical trials have demonstrated that systemic therapies may also improve the local control in breast cancer [18, 19]. Thus, there seems to be noted a recent trend of reconsideration of the importance of margin width on the incidence of local recurrences, in favour of other prognostic factors such as the biological behaviour of the tumor [15–19].

A requirement for successful BCS is a careful preoperative planning with proper localization of the lesion, especially in nonpalpable breast lesions [1]. In order to obtain adequate excisions, margins assessment techniques are also available. Wire-guided localization, radio-guided occult lesion localization (ROLL), carbon marking, intraoperative ultrasound-guided localization, cavity shave margins, and biopsy markers are commonly used, with different results in terms of LRR. The aim of this review is to investigate how these techniques may assist the surgeon to obtain adequate resections.

## 2. What Is an Adequate Surgical Margin?

A negative SM is defined by the absence of ink in any malignant cells on histology, and the distance between the closest malignant cells and the inked surface of the surgical specimen defines the microscopic margin width (Table 1) [1]. Gage et al. and Schnitt et al. have described in 1996 four types of margins status: negative if >1 mm between tumor cells and the inked surface; close if ≤1 mm; positive if presence of carcinoma at the inked margin; and focally positive if carcinoma is present at the margin in 3 or fewer low-power fields. The 5-year rates of local recurrence were 3%, 2%, 28%, and 9%, respectively [5, 6].

Park et al. have analyzed in 2000 the 8-year outcome of a series of 533 stage I or II breast cancers treated by BCS, of which 490 could be classified in one of the four margin status types: for patients with negative or close margins, LRR was 7%. Patients with extensively positive margins had an LRR of 27%, while patients with focally positive margins had an LRR of 14% [7]. In 1999 Peterson showed LRRs of 8%, 10%, and 17%, respectively, for negative, focally positive, and focally close (≤2 mm) margins from a series of consecutive 1021 stage I or II breast malignancies [8]. A strong correlation between local recurrence rates and margins status has been demonstrated in a large number of other studies based on follow-up after breast-conserving surgery plus local radiotherapy [9–14], but the adequacy of microscopic margins width remains controversial. Horiguchi has reported 7 local recurrences in a series of 217 breast cancers (3.2%) treated with BCS following a 50 Gy radiation therapy, while Karasawa reported in a retrospective analysis of 348 patients who underwent BCS an LRR of 1.7%. Both of these studies considered negative SM width of 5 mm, and Horiguchi identified the microscopic SM as an independent predictive factor for local recurrence in the conserved breast [9, 10].

In 2003 Perez studied BCS outcomes in 1037 patients with T1 and 308 patients with T2 breast cancer, with a cumulative LRR of 5.8% (78/1345) based on a threshold distance for negative/close margins equal to 3 mm, although margins status was not found to be a predictor of ipsilateral breast relapse. A higher LRR was rather noted in patients younger than 40 years with extensive intraductal component (EIC) [11]. Santiago et al. showed in 937 women with stage I or II breast cancer LRRs of 12.2% (78/639, excluded 298 patients in which the final status of margins was unknown), considering close SM ≤2 mm [12]. Another study by Karasawa et al. performed on a Japanese multicentre survey in 2005 demonstrated a crude LRR of 3.4% for patients with equal or less than 2 mm margins and an LRR of 6.3% in those with 2.1–5 mm margins [13]. Other authors, besides Gage and Park, have reported LRRs on a threshold distance for close margins ≤1 mm, such as Kreike et al. who described in a series of 1024 patients (741 with known SM width) LRRs of 11.5% [14].

Houssami et al. reported in a meta-analysis of 21 retrospective studies that the presence of positive or close SM increases the odds of local recurrences relative to negative margins (OR 2.02, *P* < 0.001), but these odds are not associated with the margins width. Thus, there is not a statistically significant difference on LRR between a margin distance of 5 mm and 1 mm. However an evident association between the odds of local recurrences and the decreasing of threshold distances for negative margins was observed, confirming the influence of SM status on LRR [15].

## 3. What Influences Margins Status?

Preoperative predicting of the SM status has recently gained a key role in planning BCS, and some predictive factors of positive margins have been described (Table 2). According to Tartter et al., a preoperative diagnosis by fine needle aspiration, a small tumor size, and the absence of DCIS or the absence of an extensive intraductal carcinoma are all associated with a decreased risk of involved margins on surgical specimen [20]. In a study based on data collected from 1648 patients through a breast cancer screening program in Melbourne, Kurniawan has identified mammographic microcalcifications (*P* < 0.0001), presence of DCIS (*P* < 0.0001), high tumor grade, multifocal disease, and lobular histology (*P* = 0.005) as factors correlated with positive margins [21]. Reedijk et al. in a prospective study of 305 patients with nonpalpable breast lesions have reported that stereotactic versus sonographic localization (*P* < 0.0001), presence of DCIS, multifocal disease, and larger tumor size (>2 cm versus <1 cm, *P* < 0.0001) are independent predictors of positive margins in BCS [22]. Shin et al. have developed a nomogram for predicting positive margins based on data collected from 1,034 patients, identifying microcalcifications on mammography, grade of mammographic density, >0.5 cm difference in tumor size between MRI and US, DCIS, and presence of lobular components on preoperative biopsy as independent predictive factors of involved margins [23].

## 4. What about DCIS?

Ductal carcinoma in situ represents 25–30% of all diagnosed breast malignancies, and its treatment with BCS has increased over the past decades [24]. Since DCIS is frequently a multifocal disease with a difficult surgical evaluation of its limits, the adequacy of SM in DCIS has gained a crucial importance and its definition remains controversial. Silverstein et al. recommended in a retrospective study of 469 specimens of DCIS a margin width of minimum 10 mm if radiotherapy is not performed, but radiotherapy for margins width less than 1 mm can be considered mandatory [25]. Rudloff et al. reported in a retrospective study of 291 women with DCIS who underwent BCS 10-year actuarial LRRs of 28%, 21%, and 19% for SM <1 mm, 1–9 mm, and ≥10 mm, respectively, without radiotherapy; these LRRs were reduced by radiotherapy [26].

Vicini et al. studied in a series of 146 DCIS patients treated with BCS a 10-year actuarial rate of recurrence equal to 12.4% and identified margins of excision >5 mm or negative (>2 mm) on reexcision as factors of decreasing risk for local recurrence, while a total volume of excision <60 cm^3^ or a tumor size ≥0.7 cm was correlated with higher LRRs. These data suggested that the adequacy of DCIS removal should be based on margins status together with volume of resection and tumor size [27]. In a recent meta-analysis of 21 studies, for a total of 7564 patients affected by DCIS, Wang et al. have demonstrated a reduced risk of ipsilateral local recurrence if tumor resected with at least 10 mm of negative margin, compared with a margin of 2 mm [28]. Therefore, there seems to be noted a general agreement on the need for relatively large margins for DCIS, especially if adjuvant radiotherapy is not performed.

## 5. Oncoplastic Surgery

Oncoplastic surgery refers to a group of surgical techniques that combine primary tumor excision with plastic surgery techniques, and it allows to achieve good cosmetic outcomes also if wider excision is performed [29]. After resection of a breast cancer a correction of a small defect may be necessary, with basic techniques of local volume replacement, more complex reconstruction techniques and may be needed to correct larger defects [29]. A common oncoplastic technique, ideal for tumors adjacent but not attached to the nipple areolar complex, is the batwing mastopexy lumpectomy, in which two half-circle incisions are made with angled wings to each side of the areola, with subsequent excision of the lesion and advance of the superior breast tissue to close the defect [30].

Another common technique is lumpectomy with reduction mammoplasty, particularly useful for tumors in large and ptotic breasts. Of note, this technique requires a careful preoperative localization of the lesion, with an exact evaluation of its extent [31].

Oncoplastic surgery is linked with a double connection with the question of margins: in fact, it allows to obtain excision with wider margins, but on the other hand, it is often difficult to determine exactly the reexcision site if a positive margin is encountered on histopathological examination, due to the handling of breast tissue to correct volume defects. In these cases, completion mastectomy is often required [29]. Interestingly, Down et al. have recently reported, in a study of comparison between patients who underwent BCS alone with patients treated with BCS and oncoplastic surgery, wider clear margins (6.1 mm versus 14.3 mm), larger specimen volumes (112.3 cm^3^ versus 484.5 cm^3^), and a subsequent lower reexcision rate (28.9% versus 5.4%) with the oncoplastic approach, without increase in complication rates [32].

Also Losken et al. have recently highlighted the oncological advantage of the oncoplastic surgery, publishing a meta-analysis of comparison between 3165 patients treated by BCS with oncoplastic surgery and 5495 patients treated by BCS alone. The reported positive margins rate is significantly lower in the oncoplastic group (12% versus 21%), although it should be noted that the rate of completion mastectomy is more common with oncoplastic surgery [33].

## 6. Preoperative Localization Techniques

### 6.1. Carbon Marking

Carbon marking technique is based on injection of sterile charcoal powder diluted with saline solution into the site of a nonpalpable breast lesion after a preoperative sonographic or stereotactic localization. A charcoal trail is created from the lesion to the superficial layers of the breast, leaving a tattoo on the skin. The subsequent surgical excision of the tumor is guided by the presence of the carbon suspension, which is removed with the lesion [34]. Because of the stability of the charcoal powder, a delayed surgery after the localization procedure is possible; on the contrary, methylene blue has a fast dispersion in the tissue. A potential disadvantage of carbon marking is obstruction of needle tip due to precipitation of charcoal particles [35]; moreover, foreign-body giant-cell reactions mimicking malignancy have been reported after vacuum-assisted breast biopsy with carbon marking [36]. Rose et al. reported in a comparison study between carbon marking and wire-guided excision a close or involved margins rate of 18.9% (27/143) with the former technique [37].

### 6.2. Wire-Guided Technique

Wire-guided localization consists of positioning a needle or a flexible wire into or alongside a nonpalpable breast lesion under mammographic, sonographic, or CT guidance. The mammographic approach is based on measurements of distances between the lesion and the nipple (or other reference points) performed on the two projections of the mammogram. In this way an approximative estimate of the lesion localization is made by the radiologist on the patient, who is supine or seated, and the wire is placed anteroposteriorly or parallel to the chest wall. Subsequent mammograms are then obtained in order to reposition the wire more accurately, and a confirmatory mammogram is finally obtained [38]. The sonographic approach is performed with the patient in a supine position, with the aid of a 5 mHz or higher transducer, and the wire is positioned under direct visualization [38, 39]. The CT approach requires a preliminary positioning of a wire on the skin in order to have a reference for measuring the lesion localization on slices, and the wire is then introduced. Various types of wires have been developed, such as standard needle, spinal needle, or curved-end retractable wire [38]. Although wire-guided technique is a relatively simple and cost-effective method for nonpalpable breast lesions localization, some disadvantages have been reported, above all the eventuality of wire dislodgment, which could affect an accurate intraoperative finding of the lesion [40]. It should be also remembered that this technique requires a good compliance from the patient, who has to keep the wire in position all the time long before the surgery. Clear margins obtained with wire-guided excision are reported to be 70.8–87.4% [37, 41–43].

### 6.3. Clip Marker after a Stereotactic or Sonographic Vacuum-Assisted Breast Biopsy

Positioning a biopsy clip is necessary when an occult breast lesion detected by mammography (i.e., microcalcifications), by ultrasound, or by MRI is completely removed within a breast biopsy procedure. After a vacuum-assisted breast biopsy conducted under stereotactic or sonographic guidance, a clip marker may be placed through the biopsy probe into the biopsy cavity to permit an effective and accurate preoperative or intraoperative localization, or to facilitate a follow-up of the lesion, especially after a neoadjuvant chemotherapy which could lead to a nearly complete tumor regression, with no longer clear visibility on imaging [44]. The first type of biopsy clip introduced was the radiopaque metallic marker of titanium or stainless steel, developed for stereotactic procedures [45]. Metallic markers embedded with a bioresorbable material (collagen plug of bovine origin, polylactic acid, polyglycolic acid) later appeared on market; while the metallic core of titanium guarantees long-term visibility and radiopacity, the packing plug of collagen aids for hemostasis after the biopsy procedure, reduces the risk of clip displacement by its expanding in the biopsy cavity, and allows an easy identification of the clip on ultrasound until its reabsorption in 6–8 weeks [46, 47]. Both of these types of clips may be used preoperatively for localization of the tumor by mammography or ultrasound, with the possibility of positioning a wire or marking the lesion's projections on the skin. An intraoperative localization without a wire is also possible, either with a radiography of the surgical specimen in order to assess the presence of the clip or by its direct visibility on ultrasound during the resection [44–47]. Clear margins obtained with this method are reported in 90–92% of cases [48, 49].

### 6.4. Radio-Guided Occult Lesion Localization (ROLL)

Luini et al. described in 1998 the ROLL technique, which consists of a preoperative injection of particles of colloidal human serum albumin labeled with radioactive technetium (^99m^Tc) into the tumor under sonographic or mammographic guidance. A scintigraphy scan of the breast is then obtained to check the correct inoculation of the tracer by comparison between its position and the localization of the lesion on mammograms. During the surgery, the tumor can be detected by a gamma probe, directly used by the surgeon to verify the adequacy of excision [50]. In addition, another radioactive tracer can be injected near to the tumor to be drained in the sentinel node, which can be easily identified by the gamma probe and then biopsied during the excision of the primary tumor. This technique was named “sentinel node and occult lesion localization” (SNOLL), and it requires two scintigraphy scans [51]. A potential complication of this procedure is the widespread dispersal of the isotope by accidental intraductal injection, which may cause a failure in identification of the lesion; therefore, this method has to be performed by an experienced breast surgeon [52]. Another concern with ROLL regards its cost: Medina-Franco et al. reported a total cost of $209 (USD) per each procedure versus $132 (USD) with wire-guided excision [53]. Negative margins reported with ROLL range from 75 to 93.5% in some studies [41–43, 51].

## 7. Margin Assessment Techniques

### 7.1. Ultrasound-Guided Excision

Many breast lesions are clearly visible on ultrasound (US), and thus an intraoperative sonographic localization with a high frequency (7.5 mHz) probe may be performed with a subsequent immediate positioning of a wire, injection of dye, marking on skin, or directly calibrating the excision. This procedure therefore avoids the need of a preoperative localization. An ultrasound scanning of the surgical specimen can also be done to assess the presence of the lesion and the adequacy of SM [54, 55]. However, it must be remembered that ductal carcinoma in situ rarely has a clear visibility on US [56], and since it represents an increasing number of breast malignancies, some methods for improving its visibility on ultrasound have been developed. The hematoma-directed US-guided technique consists of obtaining from the patient 2–5 mL of blood which is left to clot, and then this blood in injected through a needle near to the lesion or into the biopsy cavity if previously performed. This iatrogenically induced hematoma is visible on a 7.5 mHz probe during the surgery [57, 58]. Another technique used to enhance US visibility is the positioning of a titanium embedded with collagen clip after a breast biopsy. Krekel et al. have showed in a study on 201 excisions for nonpalpable invasive breast cancer that negative margins with US-guided lumpectomy are obtained in 89–96.2% of cases [42, 59].

A recent multicentre randomized trial named cosmetic outcome of the breast after lumpectomy treatment (COBALT) has investigated how US-guided excision of palpable breast lesions can influence the quality of resection, with negative margins and smaller volumes of resection reported in 97% of patients [60]. Subsequently these patients could avoid a reexcision, or a boost of radiotherapy, with a reduced psychological stress and a better cosmesis. The rationale for this better outcome is that sonography allows to visualize directly location and margins of the tumor, while preoperative imaging with mammography or magnetic resonance imaging is obtained with the patient being in a different position compared to that in the operating theatre [60].

### 7.2. Frozen Sections and Imprint Cytology

Frozen section analysis is performed with freezing and sectioning the surgical specimen with subsequent fixation and staining in order to have an extemporaneous assessment of margins; it takes about 30 minutes. Although this technique is extensively used by many surgeons to avoid the need of a postponed reexcision, some pitfalls have been reported, such as the occurrence of artifacts due to the freezing and thawing of adipose tissue in the specimen [61]. A different intraoperative method for margins evaluation is imprint cytology (“touch prep”), which consists of pressing each of the 6 faces of the specimen on 6 different slides so that any malignant cell on an involved margin is theoretically present on the cytology of the respective slide, because of the tendency of tumor cells to adhere on glass compared to adipocytes [61, 62]. Confusion on cytology interpretation may exist for specimens with irregular surfaces or presence of atypical cells, although some immunofluorescence stains (i.e., anti-MUC-1 or anti-E-cadherin antibodies) may aid the pathologist in identifying cancer cells on slides [63]. With frozen section analysis and imprint cytology, adequate SM is achieved in 89–91% of cases [61].

### 7.3. Cavity Shave Margins

Excision of cavity shave margins consists of resection of breast tissue from all 6 margins (anterior, posterior, superior, inferior, medial, and lateral) after the excision of the primary specimen, in the same procedure. This approach allows to precisely assess which margin is involved in order to calibrate the resection of the tumor. Kobbermann et al. have demonstrated with this technique 91.3% of negative or close margin if routinely performed. Interestingly, of the patients requiring reexcision of the tumor, no significant difference has been noted in terms of surgical localization technique [64]. Bolger et al. have recently reported with cavity shave margins a reexcision rate of 25%, compared with 34% if no margins assessment is carried out. Thus, cavity shave margins reduced significantly the likelihood of having residual disease (*P* = 0.02). Of note, close margins (<2 mm) are correlated with the presence of residual disease (*P* = 0.01) [65]. Marudanayagam et al. showed negative margins in 94.4% of 394 patients who underwent lumpectomy plus cavity shave margins, compared to 87.5% of 392 patients with lumpectomy only [66]. Although this technique is cost effective and it significantly reduces the rate of positive margins, it may lengthen the operating time, but it is not correlated with a worse cosmetic outcome due to larger final volumes of resection [67].

## 8. Discussion

The adequacy of SM in BCS still remains a crucial point of controversy, ranging from 10 mm for DCIS to 1–5 mm for invasive cancers [15, 25–28]. Singletary et al. stated in a review published in 2002 that it is not clear how much SM width influences LRR, although it is unacceptable to have involved margins, because the presence of tumor cells directly at the cut edge of the specimen may not be overcome merely by adjuvant therapy [1]. Then, the importance of achieving clear margins on local recurrence rate has been discussed in relation to other clinical factors correlated with the prognosis, such as the biological behaviour of the tumor (i.e., ER+/ER− or HER2) [18, 19, 68]. The NSABP B-14 trial has demonstrated an improved local control in node-negative, ER positive breast cancer patients receiving tamoxifen, with a 10-year LRR of 4.3% compared to 14.7% if tamoxifen was not administered [18]. The NSABP B-13 trial has shown a reduction of LRR from 13.4% to 2.6% in patients with node-negative, ER negative breast cancer if chemotherapy administered [19]. Thus, there seems to be noted a recent trend of reconsideration of the role of surgery in the local control of the malignancy, with a lesser interest in margins width [16, 17]. This more balanced implementation of systemic therapy and surgery is expanding also in the therapeutic strategy for positive sentinel node, and some authors are proposing not to perform the axillary dissection after the detection of micrometastases in the sentinel node [69, 70].

In 2012, Morrow et al. asserted that margins width has no influence on LRR, since systemic therapies reduce both risks of distant metastases and of local recurrence, concluding that LRR could be more correlated with the biological features of the tumor. However, it is also highlighted that adequacy of surgical resection depends on clinical judgement, so that a wider excision could be recommended, for example, in a young woman with an extensive DCIS [16]. Of note, Jatoi has responded that, while systemic therapies may improve control on early local recurrences, late recurrences are more frequent among patients treated with BCS than those treated with mastectomy [71, 72]. Finally, while there is not statistical significance in a margin of 2 mm versus a margin of 5 mm for invasive breast cancer [15], the role of margins width for DCIS, which represents 25–30% of all diagnosed breast malignancies, remains even less clear [28]. In the 13th St. Gallen International Breast Cancer Conference 2013, it was stated that systemic therapy and excellent radiation therapy techniques could make margins width less important, but the best recommendation remains a case-by-case judgement based on clinical and biological features of the tumor [73]. Moreover, a recent position statement by the American Society of Breast Surgeons on breast cancer lumpectomy margins suggests a reexcision in the case of ink positive margins but a case-by-case decision if close (<1 mm) or focally positive margin, evaluating proper adjuvant radiotherapy and systemic therapies [74].

However, since achieving negative margins (independently of the definition of adequate margins width) remains a key point of breast cancer surgery, a precise localization of the lesion is of particular importance, especially for nonpalpable breast lesions or in case of oncoplastic approach (Table 3). Certainly, it should be highlighted that obtaining negative margins depends not only on localization method or margin assessment technique, but also on extent of the lesion, on the surgical procedure, and on the pathological handling of the specimen.

An easy and cost-effective method is carbon marking: Rose et al. reported in a comparison study a close or involved margins rate of 18.9% (27/143) and 2 (0.9%) missed lesions with carbon marking, while positive margins were encountered in 29.2% (21/72) with 3 (1%) missed lesions with wire-guided localization. These differences have been nonstatistically significant, but carbon marking resulted to be less expensive than wire-guided technique [37]. Wire-guided localization is a widely used and relatively simple technique, but some complications may be encountered, such as wire dislodgment, vasovagal episodes, or pneumothorax, and it requires a good compliance from the patient who has to keep in position the wire all the time long before the surgery. Moreover clear margins obtained with wire-guided excision are reported to be 70.8–87.4%, a lower percentage in comparison with those reported with other methods like ROLL or ultrasound-guided in many systematic reviews [37, 41–43, 53].

Negative margins reported with ROLL range from 75 to 93.5% in some studies [41–43, 51], but a Nuclear Medicine Department is required. Another concern with ROLL is the eventuality of a dispersal of the radioactive tracer causing a failure in the identification of the lesion, and thus an experienced surgeon is required [52]. Clip placement after a vacuum-assisted breast biopsy appears to be effective, especially if the intraoperative localization is performed under sonographic guidance: Nurko et al. have reported clear margins in 90% (37/41) of cases [48]. A US-visible clip marker may be positioned after breast biopsies performed under sonographic or stereotactic guidance, with positive margins encountered in 8% of cases [49]. A disadvantage in clip markers is their possible dislodgment, but the average distance between the target lesion and the clip has been found to be <10 mm in 71.3% of cases [44], with an average distance of 1.1 mm if the biopsy has been performed on US [75]. Krekel et al. have shown in a study on 201 excisions for nonpalpable invasive breast cancer negative margins in 96.2% with the aid of US-guided lumpectomy [42], while Rahusen has reported clear SM in 89% of cases with the same technique [59].

Esbona et al. have demonstrated, in a systematic review on the effectiveness of intraoperative imprint cytology (IC) and frozen section analysis (FSA) versus permanent histopathology (PH), a reexcision rate of 35%, 11% and 10% with PH, IC and FSA, respectively. The pooled sensibility resulted to be 72% for IC and 83% for FSA, with a pooled specificity of 97% and 95% for IC and FSA. An intraoperative assessment permits an immediate correction of the adequacy of excision but with an elongation of the surgery time equal to 13–27 minutes [61].

## 9. Conclusion

The effectiveness of breast-conserving therapy for treatment of early stage invasive breast malignancies has been established. Surely the adequacy of margins is a crucial issue for adjusting the volume of excision, for avoiding unnecessary resection of healthy breast parenchyma, and for a good cosmetic outcome. Thus the surgical accuracy, together with improved systemic therapy and better radiation techniques, avoids reexcisions which generally are poorly tolerated by the patients. From the literature review, no single technique proved to be better among the various ones described for achieving adequate SM, because all of them have some advantages and disadvantages, although many reviews have stated the wire-guided excision to be probably the less effective method in obtaining clear margins. According to our opinion, each surgeon should adopt his most congenial localization or margin assessment technique, based on the senologic equipe experience and on available skills and technologies. Moreover, an association of two or more methods could result in a decrease in rates of involved margins. Certainly both margins status and the biological behaviour of the malignancy contribute to local recurrence rate, and future studies are needed to ascertain the relevance of both factors.

## Figures and Tables

**Table 1:** Local recurrence rates and corresponding threshold distances for negative margins are indicated for each study.

Study	Surgical margins	Local recurrences
Horiguchi et al., 2002 [9]	5 mm	3.2%
Karasawa et al., 2003 [10]	5 mm	1.7%
Perez, 2003 [11]	3 mm	5.8%
Peterson et al., 1999 [8]	2 mm	12.8% (*)
Santiago et al., 2004 [12]	2 mm	12.2%
Karasawa et al., 2005 [13]	2 mm (2.1–5 mm)	3.4% (6.3%)
Gage et al., 1996 [5]	1 mm	10.5% (*)
Park et al., 2000 [7]	1 mm	16% (*)
Kreike et al., 2008 [14]	1 mm	11.5%

*Average percentage calculated from single LRRs for each type of margins status.

**Table 2:** Most common features associated with positive surgical margins [20–23].

Predicting factors of margin status	
Presence of DCIS	*P* < 0.0001
Multifocal disease	*P* = 0.0197
Tumor size	*P* < 0.0001
Lobular histology	*P* = 0.005
Microcalcifications on mammography	*P* < 0.0001

**Table 3:** Rates of adequate margins and main disadvantages for each technique.

Technique	Rate of adequate margins	Disadvantages
Carbon marking	81.1%	Possible foreign-body reactions mimicking malignancy on follow up; obstruction of needle tip due to charcoal precipitation.
Wire-guided	70.8–87.4%	Wire dislodgment; vasovagal episodes; pneumothorax.
ROLL	75–93.5%	Possible widespread dispersal of the tracer by accidental intraductal injection; nuclear medicine department required; for experienced surgeons; expensive.
Clip marker	90–92%	Clip migration.
US-guided	89–97%	DCIS rarely visible on US if not marked with a clip or hematoma.
Cavity shave	91.3–94.4%	Long operative times.
Imprint cytology and frozen section analysis	89–91%	Sensibility equal to 72–83%; possible difficult interpretation by pathologist due to presence of irregular specimen's surfaces or atypical cells; long operative times.
